# An Integrated Proteomics and Bioinformatics Analysis of the Anticancer Properties of RT2 Antimicrobial Peptide on Human Colon Cancer (Caco-2) Cells

**DOI:** 10.3390/molecules27041426

**Published:** 2022-02-20

**Authors:** Surachai Maijaroen, Sompong Klaynongsruang, Sittiruk Roytrakul, Monruedee Konkchaiyaphum, Lapatrada Taemaitree, Nisachon Jangpromma

**Affiliations:** 1 Department of Biochemistry, Faculty of Science, Khon Kaen University, Khon Kaen 40002, Thailand; sbyd_bc@hotmail.com (S.M.); somkly@kku.ac.th (S.K.); monrko@kku.ac.th (M.K.); 2 Protein and Proteomics Research Center for Commercial and Industrial Purposes (ProCCI), Faculty of Science, Khon Kaen University, Khon Kaen 40002, Thailand; 3 Functional Ingredients and Food Innovation Research Group, National Center for Genetic Engineering and Biotechnology, National Science and Technology Development Agency, Pathum Thani 12120, Thailand; sittiruk@biotec.or.th; 4 Department of Integrated Science, Faculty of Science, Khon Kaen University, Khon Kaen 40002, Thailand; lapata@kku.ac.th

**Keywords:** apoptosis, cancer metabolism, colorectal cancer, label-free proteomics, peptide, proliferation

## Abstract

New selective, efficacious chemotherapy agents are in demand as traditional drugs display side effects and face growing resistance upon continued administration. To this end, bioactive molecules such as peptides are attracting interest. RT2 is a cationic peptide that was used as an antimicrobial but is being repurposed for targeting cancer. In this work, we investigate the mechanism by which this peptide targets Caco-2 human colon cancer cells, one of the most prevalent and metastatic cancers. Combining label-free proteomics with bioinformatics data, our data explore over 1000 proteins to identify 133 proteins that are downregulated and 79 proteins that are upregulated upon treatment with RT2. These changes occur in a dose-dependent manner and suggest the former group are related to anticancer cell proliferation; the latter group is closely related to apoptosis levels. The mRNA levels of several genes (FGF8, PAPSS2, CDK12, LDHA, PRKCSH, CSE1L, STARD13, TLE3, and OGDHL) were quantified using RT-qPCR and were found to be in agreement with proteomic results. Collectively, the global change in Caco-2 cell protein abundance suggests that RT2 triggers multiple mechanisms, including cell proliferation reduction, apoptosis activation, and alteration of cancerous cell metabolism.

## 1. Introduction

Traditional cancer therapies such as chemo- or radiotherapy cause side effects due to their indiscriminate destruction of rapidly dividing cells, which is also a hallmark of cancer [1]. However, over time, cancer cells develop resistance to these therapies through gene mutations, metabolic changes, or epigenetic modification [2,3]. As a consequence, there is a desire to develop new chemotherapy agents, and to this end, natural bioactive compounds have gained attention due to their cell specificity and minimal toxic side effects [4]. Recently, antimicrobial peptides (AMPs) [5,6,7] were repurposed and evaluated as anticancer peptides (ACPs) [8,9,10]. The rationale behind this is that cancer cell chemoresistance mechanisms can be bypassed by the AMPs’ generic mechanism of action, cell membrane disruption [3].

In particular, RT2 (NGVQPKYRWWRWWRRWW-NH_2_), a cationic antibacterial peptide from *Crocodylus siamensis* leukocytes [5,11], was shown to have anti-inflammatory [12], anti-oxidative stress [12], and anti-proliferative effects on cancer cells [11,13]. RT2 was hypothesized to enter cancer cells through electrostatic and hydrophobic interactions with the negatively charged cell membrane, before subsequently inhibiting cell migration through downregulation of the PI3K/AKT/mTOR signaling pathway and inducing apoptosis through suppression of Bcl-2 and XIAP [13]. Although these conclusions were reached on the basis of mRNA expression levels, it would be useful to understand at the translation level, which may provide richer information on how RT2 affects cancer cells.

The goal of this study is to expand our understanding of RT2’s mechanism of action using proteomics and Caco-2 human colon cancer cell, an ideal model system due to their high metastatic characteristics and resistance to traditional chemotherapy agents [14,15]. In this work, we evaluate over 1000 proteins using proteomics and identify 133 proteins that are downregulated and 79 proteins that are upregulated upon RT2 treatment. A handful of these proteins (FGF8, PAPSS2, CDK12, LDHA, PRKCSH, CSE1L, STARD13, TLE3, and OGDHL) are then discussed and the results are verified using mRNA quantification and cellular apoptosis/proliferation assays.

## 2. Results and Discussion

### 2.1. Cytotoxic Effect of RT2 on Caco-2 Cells

**Figure 1:**
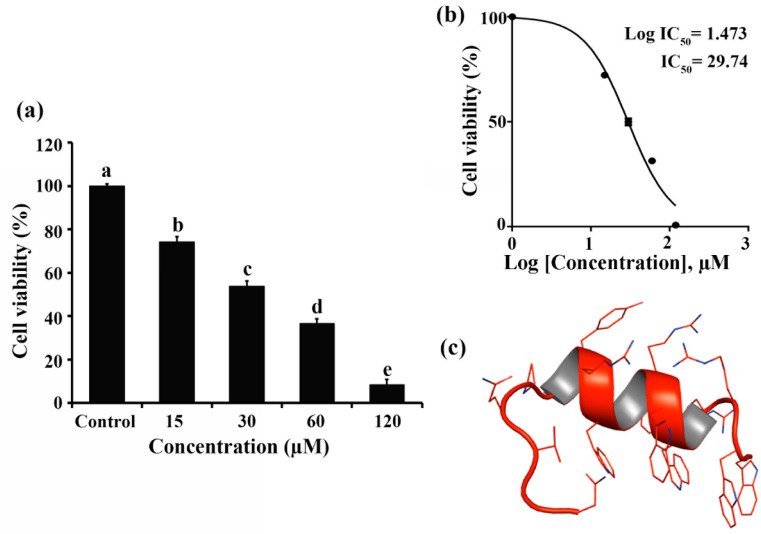
The cytotoxic effect of RT2 synthetic peptide against Caco-2 human colon cancer cells. Caco-2 cells were treated or not with increasing concentrations of RT2 (15, 30, 60, and 120 μM) for 24 h. (**a**) The percentage of cell viability was expressed as the abundance of treated Caco-2 cells relative to that of untreated Caco-2 cells. The different letter(s) on the top of each bar means there was a significant difference in the data (*p* < 0.05, *n* = 3). (**b**) The half maximal inhibitory concentration (IC_50_) value of RT2 peptide was then calculated in triplicate using GraphPad Prism version 5. (**c**) RT2 is predicted to adopt a secondary structure from its amino acid sequence (net charge of +7) using a PEP-FOLD peptide structure prediction server.

The anti-proliferative effect of RT2 against colorectal adenocarcinoma Caco-2 cells was first evaluated using the MTT assay at the concentrations used in subsequent assays (0, 15, 30, 60, and 120 μM). A significant (*p* < 0.05) dose-dependent response was observed after 24 h of treatment with cell viability of 100%, 73.2%, 52.9%, 36.4%, and 8.55% after exposure to RT2 concentrations of 0, 15, 30, 60, and 120 μM, respectively (Figure 1a). The calculated IC_50_ value (29.74 μM, Figure 1b) was found to be close to the mid-point concentration (30 μM). These observations corroborate those reported for HCT-116 colorectal carcinoma cells (RT2 dose-dependent cytotoxicity with IC_50_ value of 87.8 µg/mL or 34.49 μM) [13]. The relatively high cytotoxicity may be due to the amphipathic alpha-helix structure and cationic charge (+7) of RT2 (Figure 1c), enabling the peptide to interact with and penetrate the negatively charged cell membrane of cancer cells in an analogous manner to its mechanism of action with bacteria [5,16]. Interestingly this mechanism may be selective for cancer cells over normal cells due to their overexpression of phosphatidylserine on the inner membrane leaflet [17]. In our previous studies, 15 and 30 μM RT2 exhibited no cytotoxicity in non-cancerous Vero cells, while a slight decrease in cell viability was observed when 60 μM RT2 was used (the cell viability > 80%) [13]. Therefore, similar doses were used in the following experiments.

### 2.2. Caco-2 Cell Label-Free Based Proteomics Profiles

Previous studies reported that peptides could attack the mitochondrial membrane in addition to the cancer cell membrane [17]. Therefore, this could disrupt metabolic processes which would be observed at the proteome level [18]. Thus, assessing the effects of RT2 peptides on the proteomics changes in colon cancer cells will enable us to gain a better understanding of the mechanisms underlying the anticancer mechanisms of RT2.

To this end, label-free proteomics was performed to identify and quantify the effect of RT2 on Caco-2 cell protein expression. Cells were treated with four different doses of RT2 (0, 15, 30, and 60 µM), and the LC-MS/MS spectra obtained were matched to the protein sequences in the Uniprot Database (Homo sapiens). A total of 1044 proteins were identified from the four different treatment groups (Table S1).

**Figure 2:**
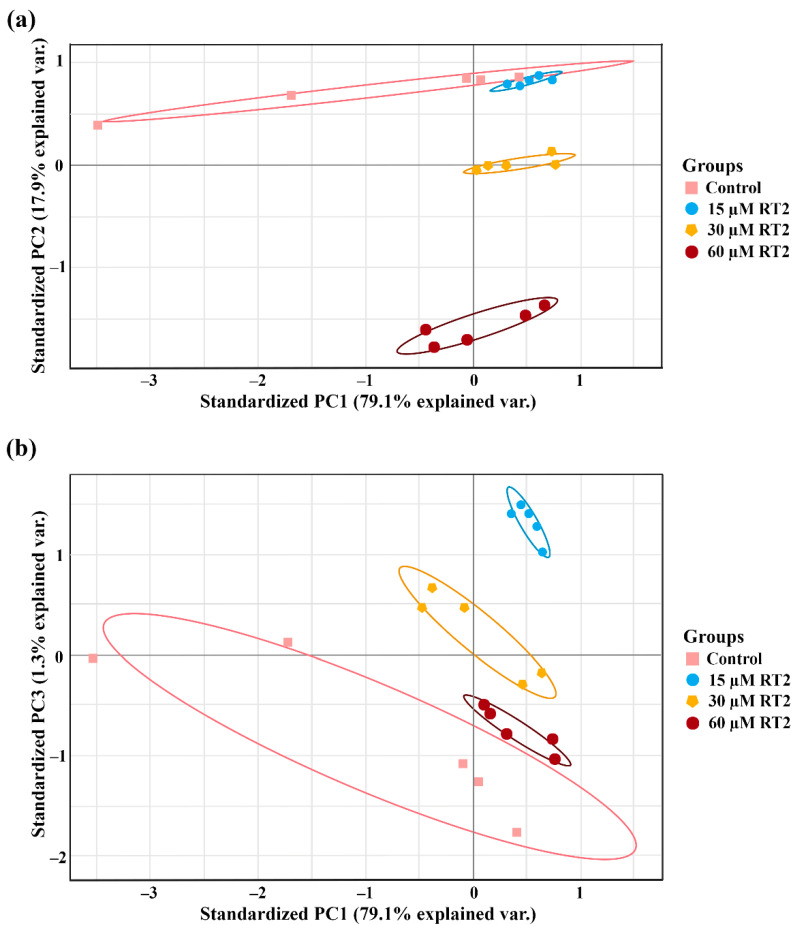
The biplot-principal component analysis (PCA) showing (**a**) PC1 vs. PC2 and (**b**) PC1 vs. PC3 of the Caco-2 proteome. The groups of datasets that are close together are highly correlated in terms of the protein expression profile for each condition. On the other hand, the groups of datasets that are far apart are less correlated. Different groups are indicated by circles, including the untreated control group, and 15, 30, and 60 µM of RT2 peptide-treated groups. Each point corresponds to a different experimental repeat.

The significant differences between different treatment groups, and in particular relative to the control untreated group, was assessed through a multivariate analysis of proteomics data using principal component analysis (PCA) of the normalized abundant proteome expression profiles (Figure 2). From this analysis, PC1 and PC2 separated the treatment groups (Figure 2a), while PC1 and PC3 completely separated these four groups (Figure 2b). The control group was clearly associated with different biochemical processes to the treatment groups, as revealed by their distinct clusters in the biplot-PCA.

For the 1044 identified, the gene-ontology PANTHER classification system algorithm (http://www.pantherdb.org; accessed on 15 September 2021) was used to provide an overview of their functions to provide insight into the biological processes and cellular components the proteins are involved in (Figure S1, see Supplementary Materials). The proteins were assigned into 18 biological functional groups: mainly cellular process (26.9%), metabolic process (13.4%), cellular component organization or biogenesis (12.7%), and biological regulation (12.3%) (Figure S1a). The identification of protein functions in terms of the cellular component was broadly associated with cell proteins (22.1%), cell part (22.1%), and organelle protein (15.8%) (Figure S1b). These results confirm our proteomics data covers a rich range of proteins involved in many different processes.

### 2.3. Caco-2 Cell Proteomics Profiling Based on the Percentage of Cell Viability

Next, the Multi Experiment Viewer (MeV) software was used to reduce large data dimensions to a smaller number of groups for simpler visualization and interpretation. Self-organizing tree algorithm (SOTA) analysis revealed that the expression of 133 proteins in Caco-2 cells was downregulated in treatment groups (Table S2). The relative changes of these proteins upon treatment are visualized as a heatmap using hierarchical cluster analysis in Figure S2. Interestingly, these changes were comparable to the dose-dependent increase in cellular toxicity of RT2 observed in our MTT assay (Figure 1a).

**Figure 3:**
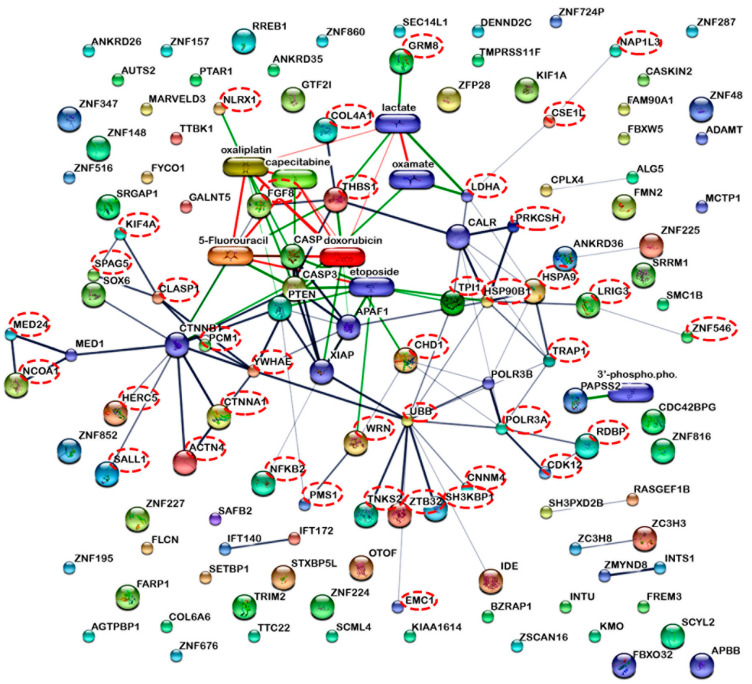
The interactions of the 133 proteins that were repressed upon treatment with RT2. This correlates with reduced cell proliferation upon RT2 treatment. Some are predicted to interact with anticancer drugs such as doxorubicin, oxaliplatin, 5-fluorouracil, and capecitabine as well as apoptotic proteins such as caspase 3 and caspase 9 using the STITCH version 5.0 software. The dashed circles indicate the filtered proteins from the proteomics dataset that interact with anticancer drugs and apoptotic proteins.

Protein-protein and protein-chemical interactions are fundamental to how cells function [19]. As a consequence, the STITCH online database was used to predict functional interactions between the 133 down-regulated proteins as well as with other known apoptotic proteins or cancer drugs such as doxorubicin, oxaliplatin, 5-fluorouracil, and capecitabine (Figure 3). In total, 122 nodes and 85 interactions edges were identified. Of the 133 proteins, 39 were predicted to have interactions with the anticancer drug and/or apoptotic proteins (dashed circles, Figure 3). These anticancer drugs were reported to inhibit colon cancer cell proliferation and enhance apoptosis during therapy [20,21,22,23]. The putative implication of some of these identified proteins is discussed in further detail below (Table 1).

Fibroblast growth factor 8 (FGF8) expression was suppressed upon RT2 treatment. From STITCH analysis, this protein is predicted to indirectly interact with anticancer drugs and known apoptotic proteins (Figure 3). More generally, FGF8 is a well-recognized oncogene that is highly expressed during the growth, proliferation, metastasis, and invasion of several cancer cells [24,25,26]. The overexpression of FGF8 in nude mice led to enhanced tumor progression [27]. Moreover, the expression of FGF8 was correlated with Wnt-1 expression and was observed to affect cancer progression in the mammary glands of mice [25]. Current work reported a significant downregulation of FGF8 in 30 and 60 µM RT2-treated Caco-2 cells. In combination, this information suggests that RT2 displays anticancer cell proliferative activity by targeting FGF8.

Bifunctional 3′-phosphoadenosine 5′-phosphosulfate synthase 2 (PAPSS2) is an enzyme involved in sulfate assimilation [28]. Sulfation is an important post-translational modification required for numerous processes such as cell adhesion, drug metabolism, and hormonal regulation [28]. For this reason, the depletion of sulfation was implicated in various pathophysiological disorders [28,29]. PAPSS2 overexpression was also suggested to induce the migration of cancer cells [29]. In this study, proteomics revealed that RT2 suppressed PAPSS2 expression in a dose-dependent manner (Table 1) which correlates with the increased cytotoxicity profile of RT2 on Caco-2 (Figure 1). Similar results were also documented in colon cancer, breast cancer, and liver cancer [28,29,30].

Our work also demonstrates that RT2 downregulates cyclin-dependent kinase 12 (CDK12) in Caco-2 cells (Table 1). CDK12 is the main transcriptional regulator of cyclin-dependent kinases that associate with cyclin K to phosphorylate the C-terminal domain of RNA polymerase II. This is necessary for transcription, elongation and translation control, DNA damage response, cellular development, growth, and differentiation [31,32,33]. The upregulation and mutation of CDK12 was documented in numerous types of cancer such as gastric, colon, and breast cancer [31,33]. Furthermore, knockdown of CDK12/cyclin K was shown to result in the accumulation of cells in the G2/M-DNA damage checkpoint, thereby arresting cell proliferation [33]. From this context, CDK12 could be a powerful biomarker and anticancer drug target, potentially for RT2 peptides [33].

Another mechanism that stimulates the growth of multiple malignancies is glycolysis, which is also a hallmark cancer cell metabolism [34,35]. Perpetual stimulation of the glycolysis pathway results in oncogene activation, with or without the suppression of tumor suppressor genes. This causes infinite malignancies proliferation [36]. Lactate dehydrogenase A chain (LDHA) is an enzyme that facilitates the final step of the glycolysis pathway through the reversible conversion of pyruvate to lactate [35]. Enhanced expression of LDHA leads to the progression of malignant cells [33,34,37] and is a tumor angiogenesis regulator [34]. On the other hand, the suppression of LDHA expression can reduce and delay tumor cells progression and formation [35]. Reduced LDHA expression can also induce cancer cells apoptosis through enhanced reactive oxygen species (ROS) levels in mitochondria and apoptosis-associated proteins such as Bax and cytochrome *c*. This mechanism of LDHA also intensifies the suppression of anti-apoptotic Bcl-2, Bcl-XL, and XIAP that leads to apoptosis induction in tumor cells [38]. These data suggest the reduction of LDHA in Caco-2 cells upon RT2 treatment might trigger apoptosis in cells and contribute to the inhibition of cell proliferation and migration.

Glucosidase 2 subunit beta or 80K-H protein (PRKCSH) is an endoplasmic reticulum (ER) glucosidase II enzyme involved in the post-translational modification of N-linked glycans [39]. The dissociation of glucose from glycoproteins by PRKCSH is necessary for protein folding and release by the endoplasmic reticulum. The overexpression of PRKCSH, therefore, causes an EGFR/RTK and PI3K/AKT pathway activation which, in turn, induce cancer cells proliferation, migration as well as apoptosis or autophagy depletion [39,40,41]. The dosage-dependent suppression of PRKCSH expression was also found in RT2-treated Caco-2 cells (Table 1), suggesting RT2 is potentially involved in the induction of cell apoptosis in a similar way.

Exportin-2 (Exp2) or chromosome segregation 1-like protein (CSE1L) is a colon-tumorigenesis-associated protein [42,43]. The current work demonstrated an increased abundance of CSE1L in untreated (control) Caco-2 cells. However, the treated Caco-2 cells with RT2 led to a significantly decreased abundance of this protein (Table 1). These observations are consistent with the report of Pimiento et al. [42] that found high CSE1L expression levels in human colon cancer cell lines such as HCT-116, HT29, and SW480 [42]. Knockdown of the gene resulted in the suppression of cell proliferation, colony formation, and triggered cell cycle arrest as well as apoptosis [42,43]. Thus, the diminished amount of CSE1L in RT2-treated Caco-2 cells might contribute to cell proliferation inhibition, as can be seen in Figure 1. In addition, a reduction in CSE1L level during RT2 exposure might contribute to apoptosis.

In summary, our proteomics and anti-proliferative data suggest the observed cytotoxicity of RT2 (Figure 1) might be associated with the decreased expression of oncogenic proteins involved in cancer cell proliferation, migration, metastasis, angiogenesis, and cell cycle development as well as apoptosis induction. In particular, the tumor suppressor proteins suggested apoptosis might be one of the main causes of reduced cell viability upon RT2 treatment. As a result, we looked to evaluate and quantify apoptosis in Caco-2 cells.

### 2.4. Apoptotic Effect of RT2 on Caco-2 Cells

**Figure 4:**
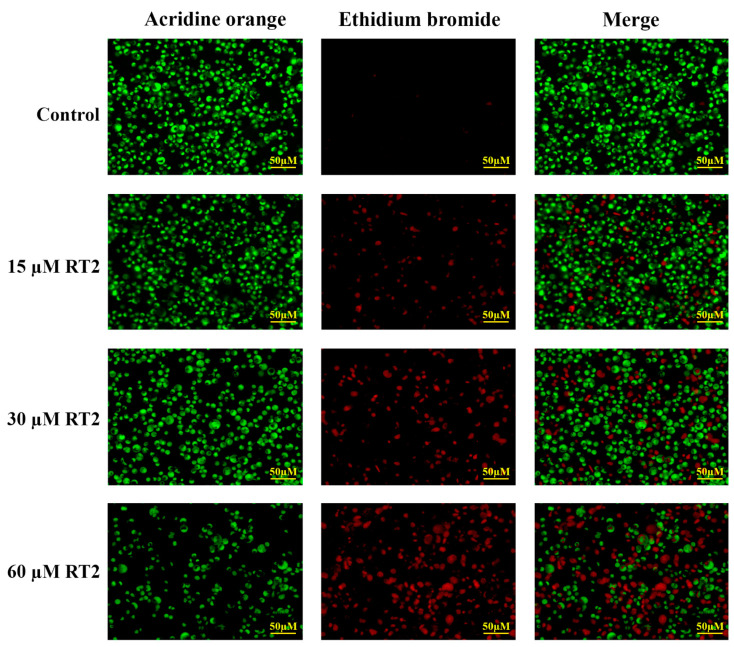
Caco-2 colon cancer cell morphological changes. Caco-2 cells were treated with increasing concentrations of RT2 synthetic peptide (0, 15, 30, and 60 µM) for 24 h and then stained with dual acridine orange/ethidium bromide (AO/EB) fluorescent dyes. Under the fluorescence microscope (magnification 20×), the live cells and the early apoptotic cells are shown in green and yellow-green, respectively. The late apoptotic and necrotic cells appeared in orange and red, respectively. The merged images show co-distribution of live, apoptotic, and necrotic cells.

To support STITCH predictions, the ability of RT2 to induce apoptosis was evaluated by staining Caco-2 cells with fluorescent dyes acridine orange (AO) and ethidium bromide (EB) (Figure 4). As can be seen from the control, when no RT2 peptide is added, the cells are healthy and viable with bright green staining from cell permeable AO. Upon treatment of the cells with RT2, more yellow-green early apoptotic cells and orange/red late apoptotic and necrotic cells could be observed due to increased uptake of cell impermeable EB through damaged cell membranes. These images suggest RT2 induces apoptosis in a dose-dependent manner.

**Figure 5:**
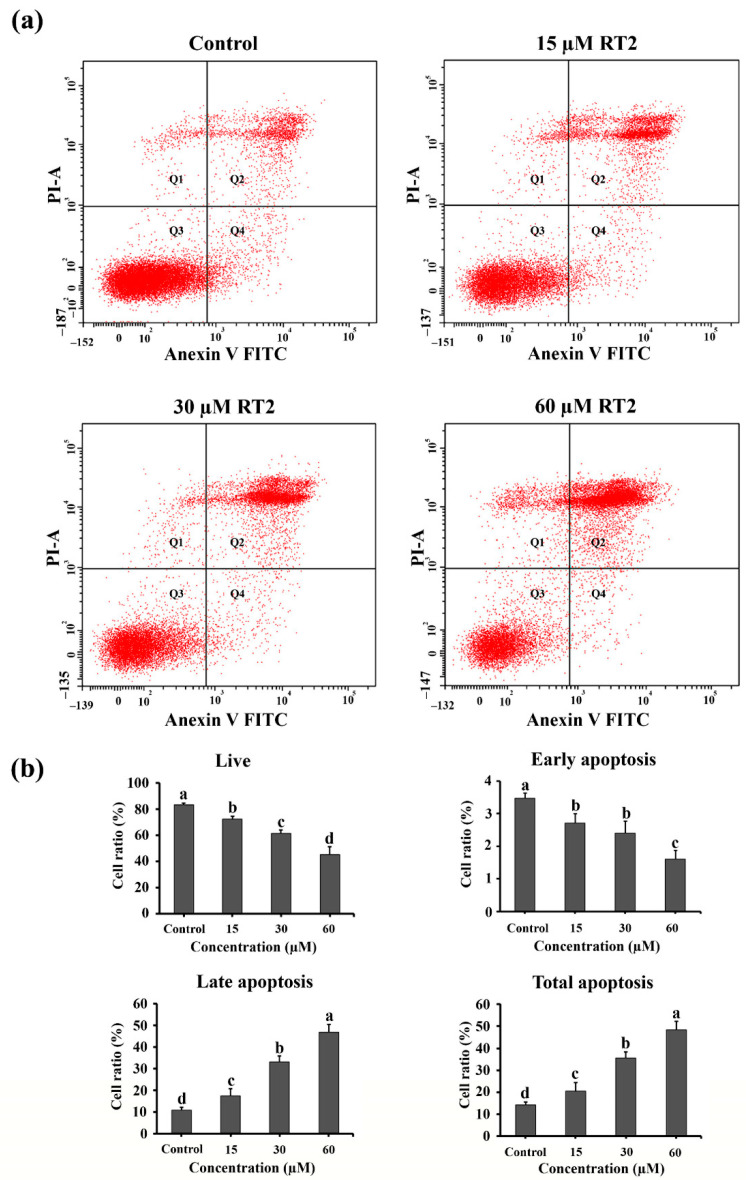
Annexin V-FITC/PI double staining coupled with flow cytometry revealed RT2 induces apoptosis in Caco-2 colon cancer cells. Cells were treated or not with increasing concentrations of RT2 (15, 30, and 60 µM) for 24 h. (**a**) The flow cytometry histograms indicate that RT2 treatment leads to an increase in the number of Caco-2 apoptotic cells. Live cells are in quadrant 3 (Q3, Annexin V-FITC negative and PI negative). Early apoptotic cells are in quadrant 4 (Q4, Annexin V-FITC positive and PI negative). Late apoptotic cells are in quadrant 2 (Q2, Annexin V-FITC positive and PI positive). Necrotic cells are in quadrant 1 (Q1, Annexin V-FITC negative and PI positive). (**b**) The bar chart quantifies the percentage of live, early, late, and total apoptotic cells. These data are represented as mean ± SD. Different letter(s) on the top of each bar are statistically different from each other (*p* < 0.05, *n* = 3).

These qualitative results were then supported by staining cells with fluorescent annexin V-FITC and propidium iodide (PI) before quantification using flow cytometry (Figure 5). Caco-2 cells could be segregated into four populations (Figure 5a) including: (1) live cells in quadrant 3 (Q3, Annexin V-FITC negative and PI negative); (2) early apoptotic cells in quadrant 4 (Q4, Annexin V-FITC positive and PI negative); (3) late apoptotic cells in quadrant 2 (Q2, Annexin V-FITC positive and PI positive); and (4) necrotic cells in quadrant 1 (Q1, Annexin V-FITC negative and PI positive). As expected from AO/EB double staining, flow cytometry demonstrated a quantitative increase in late apoptotic and necrotic cells as RT2 concentration increased (17.5, 33.2, and 46.8% for 15, 30, and 60 μM of RT2), whereas the number of living cells decreased (45.3–72.3%). At the highest concentration (60 μM of RT2), a significant (*p* < 0.05) increase in late apoptotic cells was observed. The total amount of apoptotic cells ranged from 22.2 to 48.4%, thereby demonstrating that apoptosis is an important pathway for RT2 treatment.

Several ACPs were implicated in cancer cell apoptosis such as LL-37 [44], Melittin [45], and PTP7 [46]. These membranolytic peptides may force cancer cells to undergo apoptosis by damaging the mitochondrial membrane causing the release of cytochrome *c* and thereby triggering the apoptosis pathway [16,17]. Our prior research also demonstrated that RT2 induces apoptosis in HCT-116, triggering cytochrome *c* upregulation at the mRNA level [13]. To study this apoptosis and RT2 in more detail, we re-evaluated our proteomics data set.

### 2.5. Caco-2 Cell Proteomics Profiling Based on Apoptosis Level

**Figure 6:**
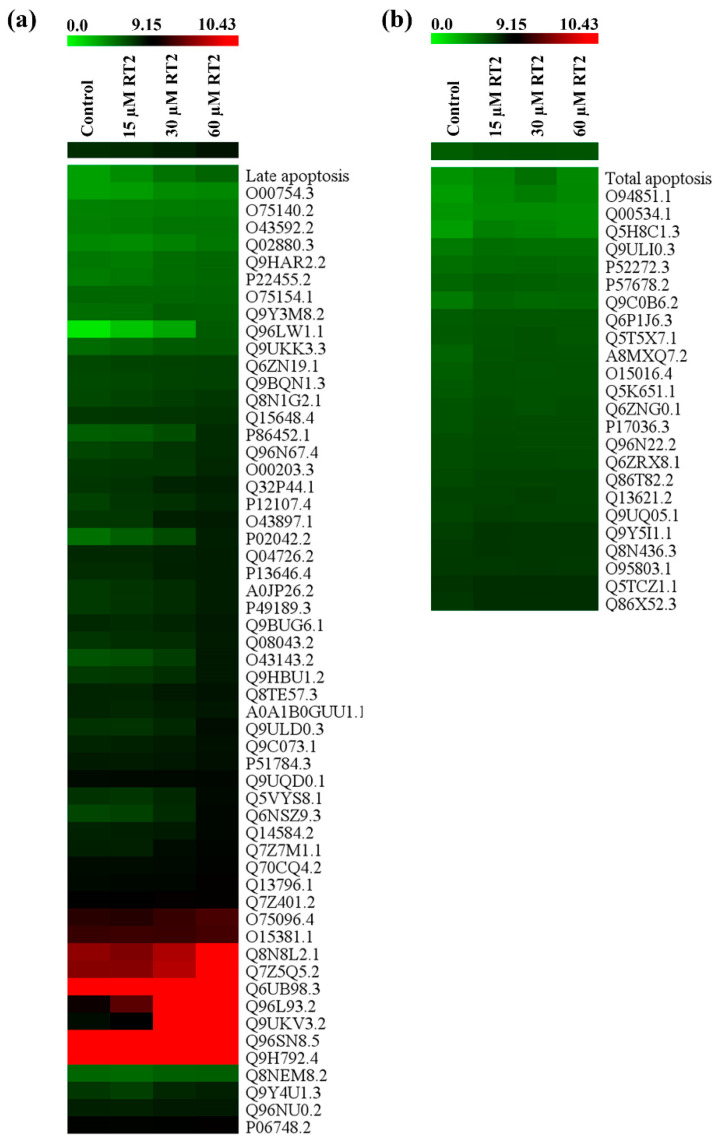
Heatmap representation of the expression levels of (**a**) 55 proteins that are enhanced in Caco-2 cells in a comparable way to late apoptosis levels and (**b**) 24 proteins that are enhanced in Caco-2 cells in a comparable way to total apoptosis levels after RT2 treatment (15, 30, and 60 µM) as analyzed by SOTA. Note that the control corresponds to untreated cells. The relative protein expression values are color coded from the lowest (green) to the highest (red).

The MeV software was used to find proteins from Caco-2 proteomics data that are upregulated and involved in apoptosis upon RT2 treatment. The cluster resulted from SOTA analysis demonstrated that an increase in expression levels of 55 proteins in RT2-treated Caco-2 cells was comparable to late apoptosis levels, while 24 proteins were correlated with total apoptosis levels as determined by flow cytometry (Table S3). The heatmap representing the hierarchical cluster analysis of the relative expression level of these proteins is shown in Figure 6. The shades of green to red indicated changes in protein expression levels from low to high when Caco-2 cells were treated with 0, 15, 30, and 60 µM of RT2.

**Figure 7:**
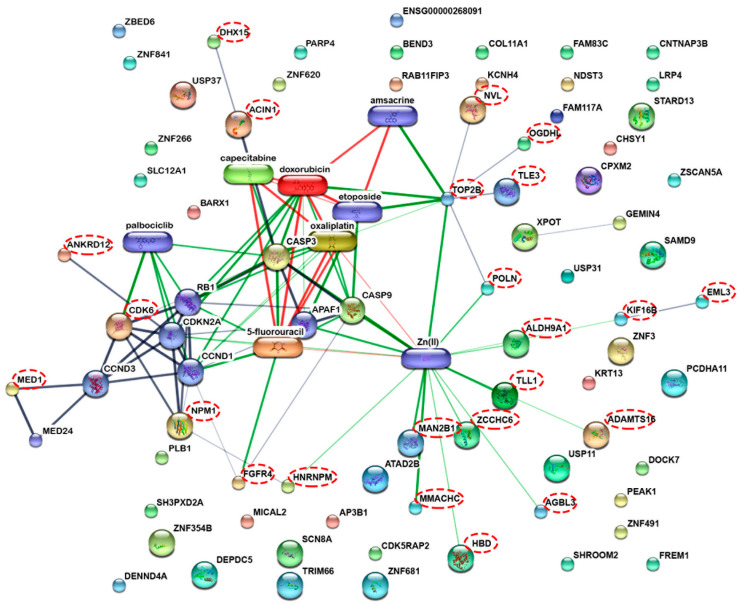
The interaction of the 79 proteins that were enhanced in their expression in a comparable way to late and total apoptosis levels and those that are predicted to associate with anticancer drugs (e.g., doxorubicin, oxaliplatin, 5-fluorouracil, and capecitabine) or apoptotic proteins (e.g., caspase 3 and caspase 9) using the STITCH version 5.0 software. The dashed circles indicate the filtered proteins from the proteomics dataset that interact with anticancer drugs and apoptotic proteins.

Of the 79 proteins identified, further functional interaction networks were investigated based on the STITCH online database predictions (Figure 7). Twenty-three proteins were predicted to interact with anticancer medicines, including doxorubicin, oxaliplatin, 5-fluorouracil, and capecitabine as well as known apoptotic proteins (dashed circles, Figure 7). The set of proteins that were enhanced upon RT2 exposure are listed in Table 2. In particular, we observed the upregulation of three proteins—STARD13, TLE3, and OGDHL, which are known to enhance apoptosis and are discussed in more detail below.

StAR-related lipid transfer protein 13 (STARD13) and its overexpression are reported to inhibit Caco-2 and HT-29 cancer cell proliferation [47]. STARD13 triggered apoptosis via the elevation of p53 tumor suppressor, BAX pro-apoptotic protein, and suppression of Bcl-2 anti-apoptotic protein. The involvement of STARD13 in apoptosis was also found in hepatocellular carcinoma cells [48] and breast cancer [49]. Our proteomic results demonstrate that RT2 enhances STARD13 expression, which in turn may be the reason Caco-2 cell apoptosis was enhanced.

Transducin-like enhancer protein 3 (TLE3) is a transcriptional co-repressor that exhibits numerous cellular functions [50]. In a colon cancer model, TLE3 repressed SW480 and Ls174t cell proliferation through the suppression of the AKT/MAPK pathway [51]. This well known signal transduction cascade results in apoptosis resistance that consequently leads to the infinite proliferation of malignancies [52]. The AKT/MAPK signaling pathway is also important in cell cycle regulation [53]. Moreover, Liu et al. [54] reported that TLE3 in colon cancer cells could degrade ring finger protein 6 (RNF6), which stimulates Wnt/β-catenin, highlighting the importance of this signaling pathway in regulating the proliferation and migration of malignancies. Similar results were found in a study of breast cancer cells from Peng et al. [50], where overexpression of TLE3 resulted in the retardation of cancer cells proliferation, migration, and also invasion [50]. Our proteomics investigation revealed that RT2 enhances TLE3 expression in Caco-2 in a dose-dependent manner (Table 2).

Another protein that enhanced expression upon RT2 treatment in a dose-dependent manner is 2-oxoglutarate dehydrogenase-like, mitochondrial (OGDHL) (Table 2). OGDHL is a tricarboxylic acid (TCA) cycle associated enzyme in the mitochondria that potentially suppresses tumors indirectly through apoptosis activation [55,56]. In cervical carcinoma cells, OGDHL overexpression can cause enhanced ROS generation resulting in oxidative stress. This triggered the release of cytochrome *c*, enhancing caspase-3 expression as well as repressing AKT signaling that inhibits the phosphorylation of NF-κB, allowing apoptosis propagation [56]. The related apoptosis induction of OGDHL was also reported to be found in the colon carcinoma cell [55]. In the presence of RT2, the increasing amount of OGDHL as a function of RT2 concentration may be linked with the observed increase in apoptosis.

### 2.6. mRNA Expression Levels of Genes Related to Cell Viability and Apoptosis Level

**Figure 8:**
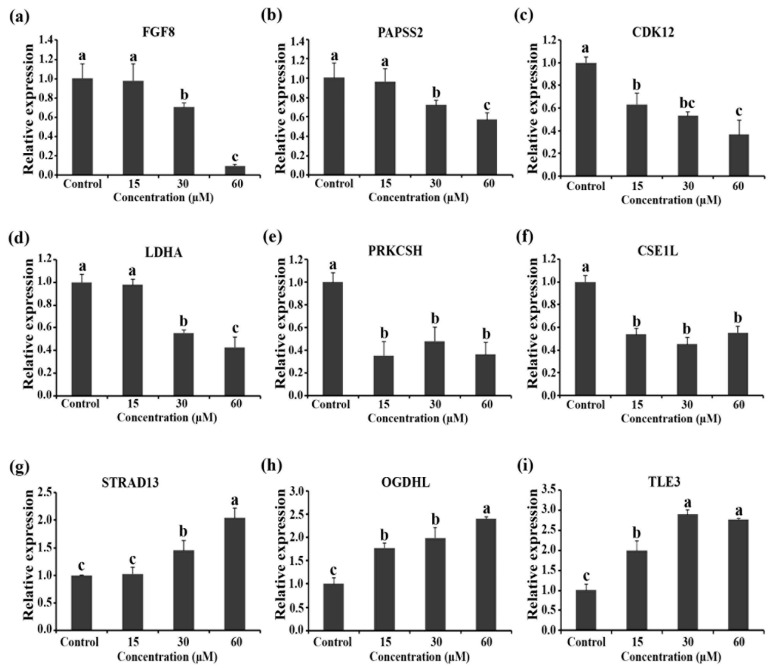
RT-qPCR results for untreated control and RT2-treated Caco-2 cells (15, 30, and 60 µM) after 24 h. (**a**–**f**) show downregulated expression of FGF8, PASS2, CDK12, LDHA, PRKCSH, and CSE1L genes, respectively. (**g**–**i**) show upregulated expression of STRAD13, OGDHL, and TLE3 genes, respectively. These data are represented as mean ± SD. Different letter(s) on the top of each bar show the statistical differences from each other (*p* < 0.05, *n* = 3).

RT-qPCR used to verify the changes in the proteome upon RT2 treatment are not experimental technique artifacts. The mRNA levels of FGF8, PASS2, CDK12, LDHA, PRKCSH, CSE1L, STRAD13, OGDHL, and TLE3 genes that were identified as being key regulators of RT2 activity, were evaluated. As expected, the expression level of anti-apoptotic/proliferation genes (FGF8, PASS2, CDK12, LDHA, PRKCSH, and CSE1L) was significantly (*p* < 0.05) decreased after the treatments with different concentrations of RT2 peptide compared to those of untreated control cells (Figure 8a–f), which is consistent with the observed protein expression changes in Table 1. These proteins are known to be highly expressed during the growth, proliferation, metastasis, and invasion of several cancer cells [24,25,26]. Our results suggest that RT2 might suppress these proteins at both the transcriptional and translational levels in Caco-2 colon cancers. On the other hand, the significant (*p* < 0.05) upregulation level of STRAD13, OGDHL, and TLE3 genes upon treatment with increasing RT2 was observed after 24 h (Figure 8g–i). This is expected based on the proteomic results (Table 2) and confirm that the upregulation of these genes is important in anti-proliferation, apoptosis activation, and the glycolysis/TCA cycle of human colon carcinoma cells.

## 3. Materials and Methods

### 3.1. RT2 Peptide Synthesis

The RT2 peptide was designed by Anunthawan et al. [57]. The sequence of this peptide is NGVQPKYRWWRWWRRWW-NH_2_ (53% hydrophobic amino acid content and +7 positive charge). RT2 was purchased at 95% purity from GL Biochem, Ltd. (Shanghai, China) using Fmoc solid-phase synthesis.

### 3.2. Cell Culture

Caco-2 human colon cancer cells were purchased from the American Type Culture Collection (ATCC, Manassas, VA, USA) were maintained in Eagle’s Minimum Essential Medium (EMEM) supplemented with 20% heat-inactivated fetal bovine serum (FBS) and 1% penicillin-streptomycin (*v*/*v*). Cells were grown and maintained in a 37 °C humidified incubator with 5% CO_2_. Every 3 days, the medium was changed, and cells were passaged at 70% confluency.

### 3.3. MTT Caco-2 Cell Proliferation and Cytotoxicity Assay

Caco-2 cells were seeded at a 1 × 10^4^ density per well onto sterile 96-well plates for 24 h. Then, the cells were treated for 24 h with different concentrations of RT2 peptides in triplicate (0, 15, 30, 60, and 120 µM). The culture medium was then discarded, and MTT dye (100 µL, final concentration of 0.5 mg/mL) was added, followed by 2 h incubation at 37 °C to yield a water-insoluble purple formazan crystal. The MTT solution was subsequently replaced with dimethyl sulfoxide (100 µL), and the plate was kept on a shaker for 5 min to solubilize the formazan salts. The absorbance at 570 nm was assessed using a Varioskan™ LUX multimode microplate reader (Thermo Fisher Scientific, Waltham, MA, USA). The percentage of cell viability or proliferation was calculated relative to the untreated control cells (100%).

### 3.4. Total Protein Extraction for Proteomic Assay

After Caco-2 cells were treated with various concentrations of RT2 (0, 15, 30, and 60 μM) for 24 h, cells were then harvested using a cell scraper and centrifuged at 3000× *g* at 4 °C for 5 min to remove the supernatant. The cells were washed with ice-cold phosphate-buffered saline (PBS) twice and subjected to protein extraction. Caco-2 cell pellets from each treatment were lysed with a trace volume of 50 mM Tris-HCl pH 7.0 containing 0.5% sodium dodecyl sulfate (SDS) buffer and then pelleted at 10,000 rpm for 15 min. The protein supernatant was collected and mixed well with 2 volumes of ice-cold acetone containing 0.1 mM dithiothreitol. After incubation overnight at −20 °C, the mixtures were centrifuged at 10,000× *g* for 15 min, and the supernatant was discarded. The pellet was dried and stored at −80 °C prior to use. To determine the concentration of protein, the pellets were resuspended in 0.5% SDS. The total soluble protein isolated from Caco-2 cells was then measured by the Lowry method with bovine serum albumin (BSA) as a standard [58].

### 3.5. In-Solution Trypsin Digestion for Proteomic Assay

In order to block the sulfhydryl group, 50 µg of protein samples were mixed with 5 mM dithiothreitol (DTT) in 10 mM ammonium bicarbonate and incubated at 60 °C for 1 h in the dark. The alkylation of sulfhydryl groups was carried out by adding 15 mM iodoacetamide (IAA) in 10 mM ammonium bicarbonate. The reaction was incubated at room temperature for 45 min in the dark. Protein samples were then digested with sequencing grade trypsin (Promega, Germany) solution (50 ng trypsin in 50% ACN/10 mM ammonium bicarbonate) at a trypsin:protein ratio of 1:20 (*w*/*w*) and incubated overnight at 37 °C. Tryptic peptides were dried at 44 °C under vacuum, protonated with 0.1% formic acid, and then injected into liquid chromatography with tandem mass spectrometry (LC-MS/MS).

### 3.6. Label-Free LC-MS/MS-Based Proteomics

LC-MS/MS analysis of tryptic peptides mixtures was performed using a Waters SYNAPT™ HDMS™ system (Waters Corp., Milford, MA, USA). The 1D-nanoscale LC was constructed with a NanoAcquity UPLC system (Waters Corp., Milford, MA, USA). In total, 4 µL of digested peptides were injected onto the reversed-phase analytical column (20 cm × 75 μm) packed with a 1.7 μm Bridged Ethyl Hybrid (BEH) C18 material (Waters Corp., Milford, MA, USA). Peptides were eluted with a linear gradient from 2% to 40% acetonitrile developed over 60 min at a flow rate of 350 nL/min. This was followed by a 15 min period of 80% acetonitrile to clean the column before returning to 2% acetonitrile for the next sample. The effluent samples were electrosprayed into a mass spectrometer (Synapt HDMS) for MS/MS analysis of peptides. Argon gas was used in the collision cell to obtain MS/MS data. MS/MS spectra obtained were processed using Maxent 3, a deconvolution software for peptides (Ensemble 1, Iterations 50, auto peak width determination) within MassLynx 4.0. The experiment was performed in 5 replicates.

### 3.7. Protein Quantitation and Identification

The DeCyder MS Differential Analysis software (DeCyderMS, GE Healthcare, Sweden) was used for protein quantitation [59]. The acquired LC-MS raw data were converted. The PepDetect module was used for automated peptide detection, charge state assignments, and quantitation based on the peptide ions signal intensities in MS mode. The analyzed MS/MS data from DeCyderMS were submitted to a database search using the Mascot software (Matrix Science, London, UK) [60]. The data was acquired from the NCBI database for protein identification. Database interrogation was; taxonomy (Homo sapiens); enzyme (trypsin); variable modifications (carbamidomethyl, oxidation of methionine residues); mass values (monoisotopic); protein mass (unrestricted); peptide mass tolerance (1 Da); fragment mass tolerance (±0.4 Da), peptide charge state (1+, 2+ and 3+), max missed cleavages (1), and instrument (ESI-Q-TOF). The relative quantitation values of each sample were displayed as a fold change. Proteins considered as identified proteins had at least one peptide with an individual mascot score corresponding to *p* < 0.05.

### 3.8. Proteomics Data Bioinformatics Analysis

The Multi Experiment Viewer (MeV) software version 4.6.1 [61] was used to normalize and quantify the alteration of protein abundance between each experimental group. The peptide intensities of Caco-2 cells from the untreated control group and RT2 treated groups from the LC-MS analyses were transformed and normalized according to the procedure of mean central tendency. Green, black, and red colors represent proteins with low, average, and high levels of expression, respectively. The analysis of variance (ANOVA) (*p* ≤ 0.05) was used to statistically analyze the significant alterations in protein expression between the experimental groups. Biplot-principal component analysis (PCA) was conducted from normalized abundant proteins expression profiles by MeV [61,62]. Additionally, gene ontology (GO), including biological process and cellular components, was carried out by PANTHER (Protein ANalysis THrough Evolutionary Relationships) classification system (http://www.pantherdb.org; accessed on 15 September 2021) [63]. The interaction analysis between proteins and proteins or proteins and small molecules/chemicals of proteomics data was performed using STITCH 5.0 database (http://stitch.embl.de/; accessed on 8 October 2021) to understand the possible interaction regulators of proteins of interest [64].

### 3.9. Apoptosis Fluorescence Morphological Assay

The ability of RT2 peptide to induce the changes in Caco-2 morphology related to apoptosis was examined by dual acridine orange/ethidium bromide (AO/EB) fluorescent staining coupled with fluorescence microscopy inspection [13]. The initial Caco-2 cells with a seeding density of 3 × 10^4^ cells per well on sterile 48-well plates were used in this experiment. After initial seeding for 24 h, the cells were subsequently treated for 24 h with RT2 peptide at final concentrations of 0, 15, 30, and 60 µM. Trypsinization was then performed by adding 50 μL of trypsin into each well to remove adherent cells from a culture plate. The 25 μL of detached cells from each treatment was gently mixed with 1 μL of dual dye solution (100 μg/mL AO and 100 μg/mL EB) on glass slides and covered by a coverslip. Afterward, inverted fluorescence microscopy (Carl Zeiss Microscopy, White Plains, NY, USA) was used to capture the change in cell morphology related to apoptosis that occurs upon RT2 treatment.

### 3.10. Apoptosis Flow Cytometric Assay

Annexin V-FITC/PI double staining (BioLegend, San Diego, CA, USA) was used to quantitatively assay apoptotic Caco-2 cells. According to the detection kit protocol, Caco-2 cells were seeded at 3 × 10^5^ density per well onto sterile 12-well plates. After a 24 h culture, 0, 15, 30, and 60 μM of RT2 were added and continually incubated for 24 h. Cells were then trypsinized and washed with ice-cold phosphate-buffered saline (PBS) twice. The detached cells were suspended in Annexin V binding buffer and stained with Annexin V-FITC/PI (BioLegend, San Diego, CA, USA). After gently mixing, the cells were then incubated for 15 min at room temperature in the dark. The stained cells were then immediately processed using a BD FACSCanto II Flow Cytometer (BD Biosciences, San Jose, CA, USA), and the data were analyzed using BD Accuri C6 software.

### 3.11. RT-qPCR Assay

Caco-2 cells were seeded on a 12-well plate at a concentration of 3×10^5^ cells/well for 24 h before treatment with RT2 peptide (0, 15, 30, and 60 µM) in at least triplicate for 24 h. Total cellular RNA of Caco-2 cells from each treatment was then isolated according to the Trizol reagent protocol (Invitrogen, CA, USA). The cDNA synthesis was conducted using the Transcriptor First Strand cDNA Synthesis kit (Fermentas, MA, USA), following the manufacturer’s instructions. RNA expression levels were evaluated by quantitative RT-PCR using the SYBR^®^ Green PCR master mix (Roche, Little Falls, NJ, USA) by a LightCycler^®^ 480 real-time PCR system (Roche, Rotkreuz, Switzerland). The well known housekeeping gene, glyceraldehyde-3-phosphate dehydrogenase (GAPDH), was used to normalize the mRNA levels of each gene between different Caco-2 samples. The primers used in the assay are listed in Table 3. The thermocycling condition for the real-time PCR was 95 °C for 3 min, followed by 40 cycles of 95 °C for 20 s, primer annealing temperatures depended on each primer (57–60 °C) for 20 s, and extension at 72 °C for 30 s. Data were presented as the relative expression of each target gene and calculated by the comparative 2^−∆∆CT^ method [65].

## 4. Conclusions

This study enriches our understanding of how RT2 inhibits human colon carcinoma Caco-2 cell proliferation at the proteome level. A total of 1044 proteins were successfully identified by label-free LC-MS/MS-based proteomics. Bioinformatics analysis of the data set identified 133 proteins that were downregulated and may be related to cell proliferation which was also decreased in a dose-dependent manner (see MTT assay). Particularly interesting proteins from this set include FGF8, PAPSS2, CDK12, LDHA, PRKCSH, and CSE1L; the main putative roles of these proteins are in anticancer cell proliferation and apoptosis induction. Subsequent AO/EB fluorescence microscopy and annexin V-FITC/PI-stained flow cytometry demonstrated that RT2 induces apoptosis in a dose-dependent manner. In addition, an increase in abundance of STARD13, TLE3, and OGDHL was observed as RT2 concentration increased; this correlates with the ability of RT2 to increase late and total apoptosis and the function of these genes. The proteomic changes were corroborated independently at the mRNA transcriptional level using RT-qPCR, which confirmed that RT2 affects both transcriptional and translation levels of these mRNAs and proteins. In summary, this study describes a new set of genes at both the proteomes and mRNA levels that are involved in anti-proliferation and apoptosis activation in human colon carcinoma cells. This supports the conclusions of previous work, which described other genes that also inhibit cancer cell growth.

## Figures and Tables

**Table 1:** Examples of proteins that were downregulated similarly to the percentage of cell viability after the RT2 treatment.

Accession	Protein Name	Gene Name	Anova (*p*)	q Value	Treatment Effect (Fold Change)		
					Caco-2 Treated with 15 µM RT2 Compared to Untreated Control	Caco-2 Treated with 30 µM RT2 Compared to Untreated Control	Caco-2 Treated with 60 µM RT2 Compared to Untreated Control
P55075.1	Fibroblast growth factor 8 (FGF-8)	FGF8	0.02300	0.00137	+1.03	−1.23	−1.11
O95340.2	Bifunctional 3′-phosphoadenosine 5′-phosphosulfate synthase 2	PAPSS2	0.05516	0.00233	−1.00	−1.17	−1.32
Q9NYV4.2	Cyclin-dependent kinase 12	CDK12	0.07786	0.00298	−1.05	−1.10	−1.15
P00338.2	L-lactate dehydrogenase A chain	LDHA	0.00001	0.00000	+1.15	−1.25	−1.18
P14314.2	Glucosidase 2 subunit beta (80K-H protein)	PRKCSH	0.00128	0.00018	−1.24	−1.23	−1.74
P55060.3	Exportin-2 (Exp2) (Cellular apoptosis susceptibility protein)	CSE1L	0.00395	0.00043	+1.06	−1.08	−1.14
Q9Y6X0.3	SET-binding protein (SEB)	SETBP1	0.00002	0.00001	−1.08	−1.15	−1.33
O95239.3	Chromosome-associated kinesin KIF4A (Chromokinesin-A)	KIF4A4	0.00600	0.00056	−1.10	−1.19	−1.33
P62258.1	14-3-3 protein epsilon (14-3-3E)	YWHAE	0.00190	0.00024	−1.09	−1.12	−1.18
Q969U6.1	F-box/WD repeat-containing protein 5	FBXW5	0.13075	0.00436	+1.03	−1.10	−1.05
P0CG47.1	Polyubiquitin-B [Cleaved into: Ubiquitin]	UBB	0.00432	0.00046	−1.09	−1.17	−1.24
Q15788.3	Nuclear receptor coactivator 1 (NCoA-1)	NCOA1	0.00007	0.00002	+1.14	−1.39	−1.21
O14646.2	Chromodomain-helicase-DNA-binding protein 1	CHD1	0.00843	0.00072	−1.14	−1.11	−1.28
Q92766.3	Ras-responsive element-binding protein 1	RREB1	0.00040	0.00008	−1.05	−1.13	−1.18
P60174.3	Triosephosphate isomerase	TPI1	0.00639	0.00059	−1.04	−1.16	−1.20
Q96R06.2	Sperm-associated antigen 5 (Astrin)	SPAG5	0.00599	0.00056	−1.07	−1.11	−1.17
O15229.2	Kynurenine 3-monooxygenase	KMO	0.00882	0.00074	−1.08	−1.09	−1.16
O43707.2	Alpha-actinin-4 (Non-muscle alpha-actinin 4)	ACTN4	0.00265	0.00031	+1.01	−1.13	−1.11
Q9H2K2.1	Poly [ADP-ribose] polymerase tankyrase-2	TNKS2	0.37048	0.01071	−1.05	−1.08	−1.09
Q9Y4B5.5	Microtubule cross-linking factor 1	MTCL1	0.05532	0.00234	−1.05	−1.09	−1.15

+: Proteins were upregulated; −: Proteins were downregulated.

**Table 2:** Examples of proteins that were upregulated and correlate with enhanced late and total apoptosis levels after RT2 treatment.

Accession	Protein Name	Gene Name	Anova (*p*)	q Value	Treatment Effect (Fold Change)		
					Caco-2 Treated with 15 µM RT2 Compared to Untreated Control	Caco-2 Treated with 30 µM RT2 Compared to Untreated Control	Caco-2 Treated with 60 µM RT2 Compared to Untreated Control
Q9Y3M8.2	StAR-related lipid transfer protein 13	STARD13	0.01026	0.00083	+1.03	+1.51	+1.38
Q9ULD0.3	2-oxoglutarate dehydrogenase-like, mitochondrial	OGDHL	0.00001	0.00000	−1.02	+1.25	+2.20
Q04726.2	Transducin-like enhancer protein 3	TLE3	0.00093	0.00014	−1.00	+1.14	+1.27
O75140.2	GATOR complex protein DEPDC5	DEPDC5	0.05765	0.00239	+1.07	+1.10	+1.21
P86452.1	Zinc finger BED domain-containing protein 6	ZBED6	0.00000	0.00000	+1.05	+1.41	+3.18
O43143.2	Pre-mRNA-splicing factor ATP-dependent RNA helicase	DHX15	0.00000	0.00000	+1.19	+1.74	+3.90
Q9HBU1.2	Homeobox protein BarH-like 1	BARX1	0.00000	0.00000	+1.05	+1.32	+2.15
Q8TE57.3	A disintegrin and metalloproteinase with thrombospondin motifs 16	ADAMTS16	0.00173	0.00023	−1.03	+1.27	+1.45
Q13796.1	Protein Shroom2 (Apical-like protein) (Protein APXL)	SHROOM2	0.00000	0.00000	+1.06	+1.06	+1.58
Q5T5X7.1	BEN domain-containing protein 3	BEND3	0.15843	0.00510	+1.10	+1.22	+1.06
O95803.1	Bifunctional heparan sulfate N-deacetylase/ N-sulfotransferase 3	NDST3	0.39396	0.01136	+1.03	+1.08	+1.04

+: Proteins were upregulated; −: Proteins were downregulated.

**Table 3:** RT-qPCR primer sequences.

Gene	Primer Sequence (5′→3′)	Annealing	Product	Reference
		(°C)	Size (bp)	Sequence
FGF8	F_ TGTCTCCCAACAGCATGTGA	59	181	NM_033164.4
	R_ CGTCTCCACGATGAGCTTTG			
PAPSS2	F_ CAAACTTGACCACGTCCGAG	57	179	NM_004670.4
	R_ TCACGCCATCATCTAGCAGG			
CDK12	F_ AGTCCACTCCCCAGTAGGAA	57	168	NM_016507.4
	R_ ACTGAGTTCAGCTCCCAGAC			
LDHA	F_ GAACAGTGGAAAGAGTGCAGAT	57	132	NM_001165415.2
	R_ AGGACAACATGCACAACCTC			
PRKCSH	F_ CACAATTGGGCACAGGGAG	59	129	NM_001289102.1
	R_ GGCCTCACTTTGTTTCCGAT			
CSE1L	F_ GGCACAGTCACTTCACAAGTT	58	171	NM_001362762.2
	R_ CGCCAATACAAACCCCATCTT			
STRAD13	F_ GCCGAGATGTTCAGTCAGGT	57	159	NM_178006.4
	R_ CACTAGCTGATGGCGTGCTA			
OGDHL	F_ GTTTCTTCAAACGTGGATCTTGC	60	139	NM_001363523.2
	R_ ATTCCTGTCCCCCGATGAAA			
TLE3	F_ CTCGCCCTTGTCAGCTCTTA	57	200	NM_001282982.2
	R_ GTTCAGCTCCGTCATGGTGA			
GAPDH	F_ GAAATGAATGGGCAGCCGTT	59	173	NM_001256799.3
	R_ CGCCCAATACGACCAAATCAG			

## Data Availability

The data that support the results and findings of this study is available from the corresponding author upon request.

## Supplementary Materials

The following supporting information can be downloaded online. Figure S1: Gene-ontology (GO) annotation of the 1044 proteins in Caco-2 cells identified in proteomics across all treatment groups using the PANTHER classification system. The identified proteins are involved in many (**a**) biological processes and (**b**) cellular components.; Figure S2: A heatmap representing the expression of 133 proteins that decrease in Caco-2 cells in a comparable way to cell viability after 15, 30, and 60 µM RT2 treatment as analyzed by SOTA. The relative protein expression values are color coded from the lowest (green) to the highest (red).; Table S1: List of the differentially expressed proteins in untreated or RT2-treated Caco-2 cells that were identified by label-free LC-MS/MS-based proteomics. The average ion peak intensity represents the protein abundance in each treatment group, and it is calculated using a log2 transformation.; Table S2: List of 133 proteins in Caco-2 cells that were downregulated in a similar way to the percentage of cell viability after RT2 treatment. The relative quantitation values of each sample were displayed as log2 values.; Table S3: List of 79 proteins in Caco-2 cells that were upregulated in a similar way to increased late and total apoptosis levels after RT2 treatment. The relative quantitation values of each sample were displayed as log2 values.

## Sample Availability

Samples of the compounds are not available from the authors.
